# Prognostic Significance of Pretreatment Apolipoprotein A-I as a Noninvasive Biomarker in Cancer Survivors: A Meta-Analysis

**DOI:** 10.1155/2018/1034037

**Published:** 2018-10-30

**Authors:** Yi Zhang, Xianjin Yang

**Affiliations:** Department of General Surgery, The First People's Hospital of Neijiang, Neijiang, 641000 Sichuan Province, China

## Abstract

**Background:**

Numerous studies have reported the prognostic significance of serum apolipoprotein A-I (ApoA-I) in various cancers, but the results have been inconsistent. The current meta-analysis was performed to investigate the association between ApoA-I level and prognosis in human malignancies.

**Methods:**

A literature search was performed using the electronic platforms of the PubMed, Cochrane Library, Web of Science, Embase, Wanfang, and China National Knowledge Infrastructure (CNKI) databases to obtain eligible articles published up to May 20, 2018. Pooled hazard ratios (HRs) with 95% confidence intervals (95% CIs) were calculated to assess the prognostic values of the ApoA-I level in cancers using STATA 12.0 software.

**Results:**

A total of 14 studies involving 9295 patients were included. The results indicated that low ApoA-I level was significantly associated with poor overall survival (OS) (HR = 0.52, 95% CI: 0.44–0.61). Significant relationships between the ApoA-I level and OS were specifically detected in nasopharyngeal carcinoma (NPC, HR = 0.63, 95% CI: 0.54–0.73), colorectal cancer (CRC, HR = 0.48, 95% CI: 0.19–0.76), and hepatocellular carcinoma (HCC, HR = 0.46, 95% CI: 0.27–0.65). The subgroup analyses for OS also further confirmed the prognostic significance of the ApoA-I level in cancers. Moreover, lower Apo A-I was associated with unfavorable cancer-specific survival (CSS, HR: 0.47, 95% CI: 0.19–0.76) in cancers, and low ApoA-I level was clearly associated with inferior total time to recurrence (TTR, HR: 0.43, 95% CI: 0.29–0.58) in HCC, poorer locoregional recurrence-free survival (LRFS) and distant metastasis-free survival (DMFS) (HR: 0.58, 95% CI: 0.42–0.74 for LRFS; HR: 0.65, 95% CI: 0.41–0.89 for DMFS) in NPC, and shorter disease-free survival (DFS, HR: 0.64, 95% CI: 0.43–0.84) in cancers*. Conclusions*. Low ApoA-I level might be an unfavorable prognostic factor in multiple malignancies, and serum ApoA-I could serve as a noninvasive marker to predict cancer prognosis.

## 1. Introduction

Cancer is a major public health problem and causes huge burdens on developed and developing countries. It is the third leading cause of death in China and ranks second in the USA [1, 2]. Although great progress has been achieved in the diagnosis and treatment of cancer, effective treatment for many cancer patients is still lacking. In view of the current situation, there has been great interest in prognostic biomarkers because of their usefulness in predicting clinical outcomes and guiding therapy [3–8].

Previous studies have investigated the correlations between serum lipid levels and human cancers [9, 10]. Serum cholesterol, high-density lipoprotein cholesterol (HDL-C), and low-density lipoprotein cholesterol (LDL-C) were shown to be associated with the prognosis in multiple human cancers, such as colorectal cancer, liver cancer, and breast cancer [11–13]. As the major protein component of plasma HDL, apolipoprotein A-I (ApoA-I) synthesized in the liver and small intestine has been reported to be associated with clinical survival in multiple human cancers, including gastric cancer, nasopharyngeal carcinoma, and breast cancer [14–17]. However, the results on the clinical value of serum ApoA-I as a useful indicator have been debatable and inconsistent, and given the limited sample size and varied methodologies of individual studies, we therefore conducted this meta-analysis to provide a systematic evaluation of the significance of serum ApoA-I as a promising prognostic marker based on all related published data.

## 2. Materials and Methods

### 2.1. Search Strategy and Study Selection

PubMed, Cochrane Library, Web of Science, Embase, Wanfang and China National Knowledge Infrastructure (CNKI) were comprehensively searched by the end of May 20, 2018. The search terms used were “apolipoprotein A-I,” “ApoA-I,” “apolipoprotein” combining with “tumor,” “neoplasms,” “malignancy,” “carcinoma” OR “cancer.” The search language was limited to English and Chinese. The references of the retrieved articles were also checked to obtain relevant studies.

Published studies that met the following criteria were included: (1) the study investigated the survival outcomes of human malignancies with low ApoA-I level versus high ApoA-I levels, (2) a cutoff value to identify pretreatment low/high ApoA-I level was given, (3) complete information for assessment of hazard ratios (HRs) and the corresponding 95% CIs for cancer prognosis were included, and (4) all patients were divided into two groups based on serum ApoA-I level.

A study was excluded if it was a nonoriginal study (review, comments, editorial, or abstract) or no useful data was available.

### 2.2. Data Extraction and Quality Assessment

The data from all studies were independently extracted by two reviewers according to an extraction template. The general information included first author, country, year of publication, included time, total sample size, survival type, follow-up period, cutoff value, cutoff selection, treatment methods, and disease stage. The HRs along with 95% CI were directly obtained from published articles, and the multivariate analysis mode was preferred. In this meta-analysis, an HR < 1 indicated a worse prognosis for subjects with low ApoA-I. If a study considered cases with low ApoA-I as a reference, then the data were converted to HR estimations that considered cases with high ApoA-I as a reference group to reflect the impact of low ApoA-I levels on cancer patients. The Newcastle–Ottawa scale (NOS) was utilized to assess the quality of the included studies, and a study with a NOS score ≥ 6 was considered to be of high quality.

### 2.3. Statistical Analysis

All pooled analyses were conducted using STATA 12.0 software (Stata, College Station, TX, USA). For the prognostic index, e.g., the overall survival (OS), HR and corresponding 95% CI were used as the summary measure. The chi-square test and *I*^2^ statistic were used to evaluate the heterogeneity. *I*^2^ > 50% or *p* < 0.1 determined significant heterogeneity, and then the random-effect model was applied. Visual funnel diagrams and Begg's test and Egger's test were utilized to assess the potential publication bias. Sensibility analysis was performed to evaluate the robustness of the combined results. All *p* values < 0.05 were regarded as statistically significant.

## 3. Results

### 3.1. Characteristics of the Studies

**Figure 1:**
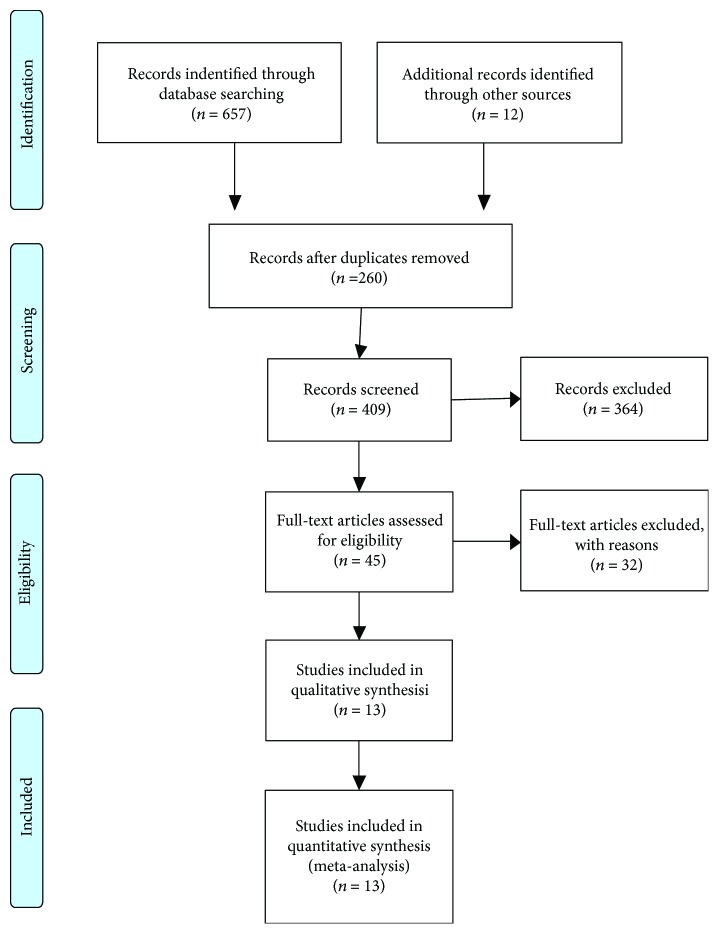
The flow chart of the literature selection.

The detailed steps involved in the literature search are shown in Figure 1. After reading the full text and further examination according to the selection criteria, ultimately, 13 publications (including 14 studies) [14–26] containing 9295 patients were included in this systematic review and meta-analysis.

All were retrospective studies, and a total of 9295 cancer patients were included from 13 studies that were carried out in China and one study from Finland. The sample size varied from 144 to 1927. With respect to prognostic outcomes, 13 studies reported OS, 1 covered disease-specific survival (DSS), 1 covered cancer-specific survival (CSS), 3 covered disease-free survival (DFS), 1 reported progression-free survival (PFS), and 2 reported total time to recurrence (TTR), locoregional recurrence-free survival (LRFS), and distant metastasis-free survival (DMFS). For calculating the prognostic values of serum ApoA-I, CSS was integrated into the meta-analysis of DSS, and PFS was integrated into the meta-analysis of DFS. Furthermore, as for cancer type, 10 different types of cancers were investigated, including non-small cell lung cancer (NSCLC); bladder cancer; gastric cancer (GC); nasopharyngeal carcinoma (NPC); breast cancer (BC); colorectal cancer (CRC); hepatocellular carcinoma (HCC); extranodal natural killer (NK)/T-cell lymphoma, nasal type (ENKTL); esophageal squamous cell carcinoma (ESCC); and renal cell carcinoma (RCC). The NOS evaluating the quality of included studies varied from 6 to 8. The main characteristics of the included studies are presented in Table 1.

### 3.2. Apolipoprotein A-I Level and OS

**Figure 2:**
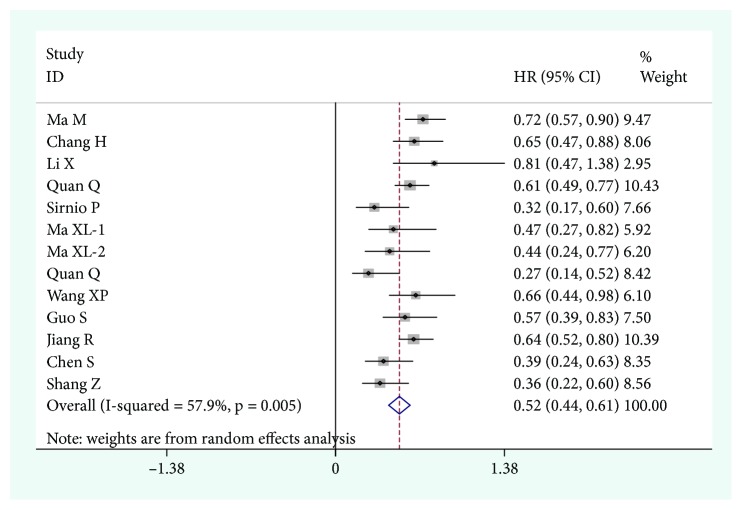
Forest plot for the relationship between ApoA-I level and OS.

A total of 13 studies with 8099 subjects was involved in the meta-analysis of OS. Because of the significant heterogeneity among studies (*I*^2^ = 57.9%, *p* = 0.005), the random-effect model was applied. As shown in Figure 2, the results indicated that the low ApoA-I group was significantly associated with shortened OS time (HR = 0.52, 95% CI: 0.44–0.61, *p* < 0.001).

### 3.3. Subgroup Analysis

The subgroup analysis for OS was conducted to further explore the correlation between ApoA-I level and various cancers. The results showed that the ApoA-I level could serve as a prognostic biomarker especially in NPC (HR = 0.63, 95% CI: 0.54–0.73, *p* < 0.001), CRC (HR = 0.48, 95% CI: 0.19–0.76, *p* < 0.001), and HCC (HR = 0.46, 95% CI: 0.27–0.65, *p* < 0.001). As shown in Table 2, the subgroup analyses were implemented based on cancer type, cutoff value selection, stages, follow-up time, analysis models, and treatments. The calculated pooled HR values were significantly less than 1.0 in those subgroup analyses, which all suggested that low ApoA-I level was a significant unfavorable prognostic factor for OS.

### 3.4. Apolipoprotein A-I Level and Secondary Outcomes

We also investigated the relationships between and serum ApoA-I level and secondary outcomes in cancer patients. The detailed results are provided in Table 3, and we found that lower Apo A-I was associated with poor CSS (HR: 0.47, 95% CI: 0.19–0.76, *p* < 0.01) in cancers, and low ApoA-I level was a prognostic factor for inferior TTR (HR: 0.43, 95% CI: 0.29–0.58, *p* < 0.01) in HCC, LRFS (HR: 0.58, 95% CI: 0.42–0.74, *p* < 0.01) and DMFS (HR: 0.65, 95% CI: 0.41–0.89, *p* < 0.01) in NPC, and DFS (HR: 0.64, 95% CI: 0.43–0.84, *p* < 0.01) in various cancers.

### 3.5. Publication Bias

**Figure 3:**
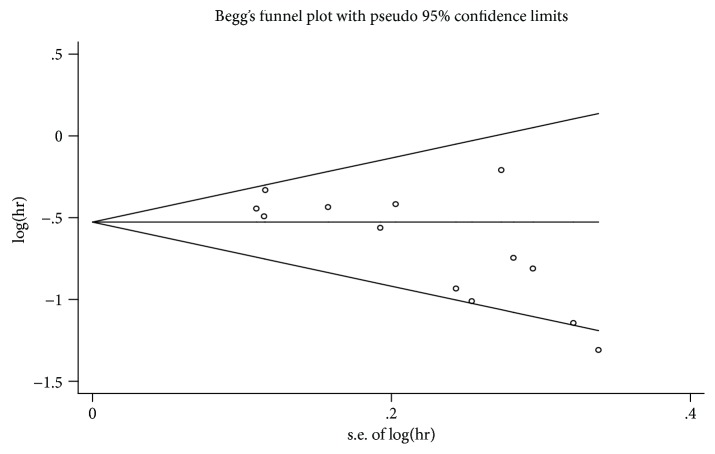
Funnel plots for publication bias test for OS.

Begg's plot is shown in Figure 3, and Begg's test and Egger's test showed that there was potential publication bias in OS (Pr_Begg's test_ > |*z*| = 0.006 (continuity corrected); Pr_Egger's test_ > |*z*| = 0.005). Then, the “trim and fill method” was also adopted, and after correction, the adjusted pooled HR was 0.555 (95% CI: 0.480–0.642, *p* < 0.001), which indicated that no significant publication bias existed.

### 3.6. Sensitivity Analysis

**Figure 4:**
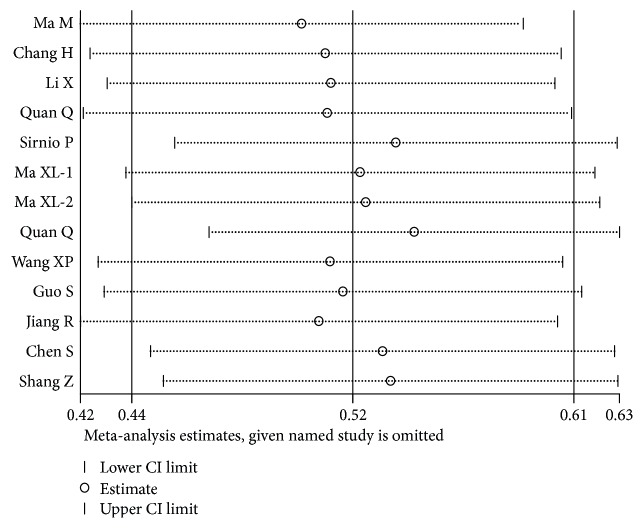
Sensitivity analysis of OS.

The results indicated that any individual study had little effect on the overall results (Figure 4), which suggested that our results were relatively stable and credible.

## 4. Discussion

ApoA-I belongs to the apolipoprotein A1/A4/E family, and it plays pivotal roles in both lipid metabolism (such as liver excretion of cholesterol, intracellular reuse of fatty acids, and as an important carrier and cofactor) and diverse human diseases [27–31]. It is well known that systemic inflammatory responses are actively involved in oncogenesis, progression, and survival prediction in cancer patients [32–34]. ApoA-I, as an indispensable component of HDL, inhibits monocyte chemotaxis and recruitment and was shown to be involved in inflammatory reactions, and ApoA-I/HDL can be activated and participate in antitumor activities in mature immune systems [31, 35]. ApoA-I can also inhibit tumor progression and protect against tumor development in vivo and in vitro. It was reported that ApoA-I established antitumor properties by interacting with C1QBP in colon cancer, and ApoA-I could also inhibit colitis-propelled carcinogenesis and modulate tumorigenicity and immunogenicity [36–39]. Studies also demonstrated the antitumorigenic effects of ApoA-I in vivo and that ApoA-I could potently suppress tumor growth and metastasis in vivo and improve survival in mouse tumor models [40, 41]. Additionally, ApoA-I exerted multiple functional effects in the tumor microenvironment [31, 41].

As far as we know, this is the first meta-analysis that has provided an updated understanding of the prognostic value of the serum ApoA-I level in various malignancies. After a synthesized literature search, a total of 13 published articles was collected involving a total of 9295 patients. From the pooled results, we found that a low level of serum ApoA-I was significantly associated with poor OS (HR = 0.52, 95% CI: 0.44–0.61) in human cancers. Furthermore, we also investigated the prognostic values of ApoA-I level in certain types of cancers and found that the pretreatment of ApoA-I level could act as a prognostic indicator in NPC (HR = 0.63, 95% CI: 0.54–0.73), CRC (HR = 0.48, 95% CI: 0.19–0.76), and HCC (HR = 0.46, 95% CI: 0.27–0.65). The subgroup analyses further revealed the prognostic significance of ApoA-I for OS in cancer patients. In addition, the relationships between serum ApoA-I level and secondary outcomes were also investigated, and the ApoA-I level was found to be associated with CSS (HR: 0.47, 95% CI: 0.19–0.76) in cancers, and the ApoA-I level might be a prognostic indicator for TTR (HR: 0.43, 95% CI: 0.29–0.58) in HCC, LRFS (HR: 0.58, 95% CI: 0.42–0.74) and DMFS (HR: 0.65, 95% CI: 0.41–0.89) in NPC, and DFS (HR: 0.64, 95% CI: 0.43–0.84) in cancers. Thus, the serum ApoA-I level might be a candidate biomarker with clinical utility in human malignant tumors.

Several limitations should be acknowledged in our meta-analysis. First, the total sample size was insufficient, and the number of studies included was also relatively small. Second, most of the participants were Chinese, and only one study was conducted with a European cohort. Third, only eligible articles published in English or Chinese were included in this meta-analysis. Fourth, potential bias might exist for OS in studies, although the “trim and fill method” was adopted and no significant bias was found. The sensitivity analysis also indicated the robustness of the pooled HR for OS. In addition, there was obvious heterogeneity for OS, and the heterogeneity could not be totally eliminated by subgroup analysis. Finally, the cutoff values for low ApoA-I level varied in different studies.

In summary, our meta-analysis showed that pretreatment low ApoA-I level was related to worse survival in patients with various tumors. Serum ApoA-I level might be a powerful and noninvasive biomarker to predict cancer prognosis. However, before ApoA-I levels are routinely applied in clinical management, large-scale and well-designed studies with unified cutoff values are necessary to validate our results.

## Figures and Tables

**Table 1:** Main characteristics of all included studies.

Study	Year	Cancer type	Country	Included time	Total sample	Follow-up	Cutoff value	Cutoff selection	Treatment methods	Stage	Survival type	NOS
Chen S	2018	NSCLC	China	2008–2010	141	≥5 years	1.17 g/l	X-tile software	Mixed	Mixed	OS	6
Shang Z	2018	Bladder cancer	China	2004–2011	470	≥5 years	1.19 g/l	ROC analysis	With surgery	No metastasis	OS, CSS	7
Ma M	2018	GC	China	2005–2010	1201	≥5 years	1.4 g/l	X-tile software	With surgery	Mixed	OS	8
Chang H	2018	NPC	China	2010–2011	1927	≥5 years	1.125 g/l	ROC analysis	No surgery	No metastasis	OS, DFS, LRFS, DMFS	8
Li X	2017	BC	China	2008–2011	1044	≥5 years	1.56 g/l	ROC analysis	With surgery	No metastasis	OS, DFS	7
Quan Q	2017	CRC	China	2005–2013	721	≥5 years	1.105 g/l	ROC analysis	No surgery	Metastasis	OS	7
Sirnio P	2017	CRC	Finland	2006–2010	144	≥5 years	1.235 g/l	ROC analysis	With surgery	Mixed	OS	6
Ma XL-1	2016	HCC	China	2012–2015	224	<5 years	1.04 g/l	X-tile software	With surgery	Mixed	OS, TTR	7
Ma XL-2	2016	HCC	China	2012-2013	219	<5 years	1.04 g/l	X-tile software	With surgery	Mixed	OS, TTR	6
Quan Q	2016	ENKTL	China	2002–2014	236	≥5 years	0.95 g/l	ROC analysis	No surgery	Mixed	OS, PFS	7
Wang XP	2016	ESCC	China	2007–2009	210	≥5 years	1.21 g/l	ROC analysis	With surgery	Mixed	OS	6
Guo S	2016	RCC	China	2000–2012	755	≥5 years	1.04 g/l	ROC analysis	With surgery	Mixed	OS, DFS	7
Luo XL	2015	NPC	China	2004–2007	1196	≥5 years	1.025 g/l	ROC analysis	No surgery	No metastasis	DSS, LRFS, DMFS	8
Jiang R	2014	NPC	China	2003–2009	807	≥5 years	1.065 g/l	ROC analysis	No surgery	Metastasis	OS	7

NSCLC: non-small cell lung cancer; GC: gastric cancer; NPC: nasopharyngeal carcinoma; BC: breast cancer; CRC: colorectal cancer; HCC: hepatocellular carcinoma; ENKTL: extranodal natural killer (NK)/T-cell lymphoma, nasal type; ESCC: esophageal squamous cell carcinoma; RCC: renal cell cancer; OS: overall survival; CSS: cancer-specific survival; TTR: time to recurrence; DFS: disease-free survival; PFS: progression-free survival; DSS: disease-specific survival; DMFS: distant metastasis-free survival; LRFS: locoregional recurrence-free survival; ROC: the receiver operating characteristic curve.

**Table 2:** Results of subgroup analysis of pooled HRs of OS.

Stratified analysis	No. of studies	Pooled HR (95% CI)	*p* value	Heterogeneity	
				*I* ^2^ (%)	P_h_
(1) Cancer type					
Gastrointestinal cancer	6	0.55 (0.43–0.68)	<0.001	52.1	0.064
Non-gastrointestinal cancer	7	0.50 (0.37–0.63)	<0.001	63.9	0.011
(2) Cutoff value selection					
ROC analysis	9	0.52 (0.41–0.63)	<0.001	62.1	0.007
X-tile software	4	0.52 (0.35–0.69)	<0.001	59.4	0.061
(3) Stage					
No metastasis	3	0.56 (0.31–0.81)	<0.001	64.7	0.059
Metastasis	2	0.63 (0.53–0.72)	<0.001	0.0	0.773
Mixed	8	0.48 (0.36–0.60)	<0.001	60.9	0.012
(4) Follow-up time					
<5 years	2	0.46 (0.27–0.65)	<0.001	0.0	0.878
≥5 years	11	0.53 (0.43–0.63)	<0.001	64.1	0.002
(5) Analysis modes					
Univariate analysis	2	0.73 (0.57–0.88)	<0.001	0.0	0.707
Multivariate analysis	11	0.49 (0.41–0.58)	<0.001	53.6	0.018
(6) Treatments					
Mixed	1	0.39 (0.24–0.63)	<0.001	—	—
No surgery	4	0.55 (0.39–0.71)	<0.001	73.1	0.011
With surgery	8	0.53 (0.40–0.65)	<0.001	52.5	0.040

**Table 3:** Analyses of secondary outcomes for ApoA-I in cancers.

Secondary outcomes	No. of studies	No. of cases	Pooled HR (95% CI)	*p* value	Heterogeneity	
					*I* ^2^ (%)	Model
CSS	2	1666	0.47 (0.19–0.76)	<0.001	77.5	Random
TTR	2	443	0.43 (0.29–0.58)	<0.001	0.0	Fixed
LRFS	2	3123	0.58 (0.42–0.74)	<0.001	0.0	Fixed
DMFS	2	3123	0.65 (0.41–0.89)	<0.001	73.0	Random
DFS	4	3962	0.64 (0.43–0.84)	<0.001	65.9	Random

## Abbreviations

ApoA-I: Apolipoprotein A-I
HDL-C: High-density lipoprotein cholesterol
LDL-C: Low-density lipoprotein cholesterol
CNKI: China National Knowledge Infrastructure
HRs: Hazard ratios
95% CIs: 95% confidence intervals
NSCLC: Non-small cell lung cancer
GC: Gastric cancer
NPC: Nasopharyngeal carcinoma
BC: Breast cancer
CRC: Colorectal cancer
HCC: Hepatocellular carcinoma
ENKTL: Extranodal natural killer (NK)/T-cell lymphoma, nasal type
ESCC: Esophageal squamous cell carcinoma
RCC: Renal cell cancer
OS: Overall survival
CSS: Cancer-specific survival
TTR: Time to recurrence
DFS: Disease-free survival
PFS: Progression-free survival
DSS: Disease-specific survival
DMFS: Distant metastasis-free survival
LRFS: Locoregional recurrence-free survival
ROC: Receiver operating characteristic curve.

## References

[1] Siegel R. L., Miller K. D., Jemal A. (2017). Cancer statistics, 2017. *CA: a Cancer Journal for Clinicians*.

[2] Chen W., Zheng R., Baade P. D. (2016). Cancer statistics in China, 2015. *CA: a Cancer Journal for Clinicians*.

[3] Zhu X., Chen F., Shao Y., Xu D., Guo J. (2017). Long intergenic non-protein coding RNA 1006 used as a potential novel biomarker of gastric cancer. *Cancer Biomarkers*.

[4] Chen M., Wu X., Ma W. (2017). Decreased expression of lncRNA VPS9D1-AS1 in gastric cancer and its clinical significance. *Cancer Biomarkers*.

[5] Liu F.T., Ou Y.X., Zhang G.P., Qiu C., Luo H.L., Zhu P.Q. (2016). *HOXB7* as a promising molecular marker for metastasis in cancers: a meta-analysis. *OncoTargets and Therapy*.

[6] Lian D., Amin B., Du D., Yan W. (2017). Enhanced expression of the long non-coding RNA SNHG16 contributes to gastric cancer progression and metastasis. *Cancer Biomarkers*.

[7] Gong W., Tian M., Qiu H., Yang Z. (2017). Elevated serum level of lncRNA-HIF1A-AS1 as a novel diagnostic predictor for worse prognosis in colorectal carcinoma. *Cancer Biomarkers*.

[8] Zhao T., Wu L., Li X., Dai H., Zhang Z. (2017). Large intergenic non-coding RNA-ROR as a potential biomarker for the diagnosis and dynamic monitoring of breast cancer. *Cancer Biomarkers*.

[9] Mancini R., Noto A., Pisanu M. E., De Vitis C., Maugeri-Saccà M., Ciliberto G. (2018). Metabolic features of cancer stem cells: the emerging role of lipid metabolism. *Oncogene*.

[10] Cruz P. M. R., Mo H., McConathy W. J., Sabnis N., Lacko A. G. (2013). The role of cholesterol metabolism and cholesterol transport in carcinogenesis: a review of scientific findings, relevant to future cancer therapeutics. *Frontiers in Pharmacology*.

[11] Zhou P., Li B., Liu B., Chen T., Xiao J. (2018). Prognostic role of serum total cholesterol and high-density lipoprotein cholesterol in cancer survivors: a systematic review and meta-analysis. *Clinica Chimica Acta*.

[12] Wang Y., Wang Z. Q., Wang F. H. (2016). Predictive value of chemotherapy-related high-density lipoprotein cholesterol (HDL) elevation in patients with colorectal cancer receiving adjuvant chemotherapy: an exploratory analysis of 851 cases. *Oncotarget*.

[13] Li X., Tang H., Wang J. (2017). The effect of preoperative serum triglycerides and high-density lipoprotein-cholesterol levels on the prognosis of breast cancer. *Breast*.

[14] Ma M., Yuan S. Q., Chen Y. M., Zhou Z. W. (2018). Preoperative apolipoprotein B/apolipoprotein A1 ratio: a novel prognostic factor for gastric cancer. *OncoTargets and Therapy*.

[15] Chang H., Wei J. W., Chen K. (2018). Apolipoprotein A-I is a prognosticator of nasopharyngeal carcinoma in the era of intensity-modulated radiotherapy. *Journal of Cancer*.

[16] Pakzad R., Safiri S. (2017). The effect of preoperative serum triglycerides and high-density lipoprotein-cholesterol levels on the prognosis of breast cancer: methodological issue. *Breast*.

[17] Quan Q., Huang Y., Chen Q. (2017). Impact of serum apolipoprotein A-I on prognosis and bevacizumab efficacy in patients with metastatic colorectal cancer: a propensity score-matched analysis. *Translational Oncology*.

[18] Sirniö P., Väyrynen J. P., Klintrup K. (2017). Decreased serum apolipoprotein A1 levels are associated with poor survival and systemic inflammatory response in colorectal cancer. *Scientific Reports*.

[19] Ma X. L., Gao X. H., Gong Z. J. (2016). Apolipoprotein A1: a novel serum biomarker for predicting the prognosis of hepatocellular carcinoma after curative resection. *Oncotarget*.

[20] Quan Q., Chen Q., Chen P. (2016). Decreased apolipoprotein A-I level indicates poor prognosis in extranodal natural killer/T-cell lymphoma, nasal type. *OncoTargets and Therapy*.

[21] Wang X. P., Li X. H., Zhang L. (2016). High level of serum apolipoprotein A-I is a favorable prognostic factor for overall survival in esophageal squamous cell carcinoma. *BMC Cancer*.

[22] Guo S., He X., Chen Q. (2016). The effect of preoperative apolipoprotein A-I on the prognosis of surgical renal cell carcinoma: a retrospective large sample study. *Medicine*.

[23] Luo X. L., Zhong G. Z., Hu L. Y. (2015). Serum apolipoprotein A-I is a novel prognostic indicator for non-metastatic nasopharyngeal carcinoma. *Oncotarget*.

[24] Jiang R., Yang Z. H., Luo D. H. (2014). Elevated apolipoprotein A-I levels are associated with favorable prognosis in metastatic nasopharyngeal carcinoma. *Medical Oncology*.

[25] Shang Z., Wang J., Wang X. (2018). Preoperative serum apolipoprotein A-I levels predict long-term survival in non-muscle-invasive bladder cancer patients. *Cancer Management and Research*.

[26] Chen S., Lai Y., He Z. (2018). Establishment and validation of a predictive nomogram model for non-small cell lung cancer patients with chronic hepatitis B viral infection. *Journal of Translational Medicine*.

[27] Yui Y., Aoyama T., Morishita H., Takahashi M., Takatsu Y., Kawai C. (1988). Serum prostacyclin stabilizing factor is identical to apolipoprotein A-I (Apo A-I). A novel function of Apo A-I. *The Journal of Clinical Investigation*.

[28] Yao X., Gordon E. M., Figueroa D. M., Barochia A. V., Levine S. J. (2016). Emerging roles of apolipoprotein E and apolipoprotein A-I in the pathogenesis and treatment of lung disease. *American Journal of Respiratory Cell and Molecular Biology*.

[29] Zhu Y. M., Verma S., Fung M., McQueen M. J., Anderson T. J., Lonn E. M. (2017). Association of apolipoproteins B and A-1 with markers of vascular health or cardiovascular events. *The Canadian Journal of Cardiology*.

[30] Mangaraj M., Nanda R., Panda S. (2016). Apolipoprotein A-I: a molecule of diverse function. *Indian Journal of Clinical Biochemistry*.

[31] Zamanian-Daryoush M., DiDonato J. A. (2015). Apolipoprotein A-I and cancer. *Frontiers in Pharmacology*.

[32] Dupré A., Malik H. Z. (2018). Inflammation and cancer: what a surgical oncologist should know. *European Journal of Surgical Oncology*.

[33] Dolan R. D., McSorley S. T., Horgan P. G., Laird B., McMillan D. C. (2017). The role of the systemic inflammatory response in predicting outcomes in patients with advanced inoperable cancer: systematic review and meta-analysis. *Critical Reviews in Oncology/Hematology*.

[34] Buchta Rosean C. M., Rutkowski M. R. (2017). The influence of the commensal microbiota on distal tumor-promoting inflammation. *Seminars in Immunology*.

[35] Iqbal A. J., Barrett T. J., Taylor L. (2016). Acute exposure to apolipoprotein A1 inhibits macrophage chemotaxis in vitro and monocyte recruitment in vivo. *Elife*.

[36] Gkouskou K. K., Ioannou M., Pavlopoulos G. A. (2016). Apolipoprotein A-I inhibits experimental colitis and colitis-propelled carcinogenesis. *Oncogene*.

[37] Medina-Echeverz J., Vasquez M., Gomar C., Ardaiz N., Berraondo P. (2015). Overexpression of apolipoprotein A-I fused to an anti-transforming growth factor beta peptide modulates the tumorigenicity and immunogenicity of mouse colon cancer cells. *Cancer Immunology, Immunotherapy*.

[38] Yi Z. F., Cho S. G., Zhao H. (2009). A novel peptide from human apolipoprotein (a) inhibits angiogenesis and tumor growth by targeting c-Src phosphorylation in VEGF-induced human umbilical endothelial cells. *International Journal of Cancer*.

[39] Kim K., Kim M. J., Kim K. H. (2017). C1QBP is upregulated in colon cancer and binds to apolipoprotein A-I. *Experimental and Therapeutic Medicine*.

[40] Su F., Kozak K. R., Imaizumi S. (2010). Apolipoprotein A-I (apoA-I) and apoA-I mimetic peptides inhibit tumor development in a mouse model of ovarian cancer. *Proceedings of the National Academy of Sciences of the United States of America*.

[41] Zamanian-Daryoush M., Lindner D., Tallant T. C. (2013). The cardioprotective protein apolipoprotein A1 promotes potent anti-tumorigenic effects. *The Journal of Biological Chemistry*.

